# Deep Brain Stimulation in the Treatment of Parkinson’s Disease

**DOI:** 10.7759/cureus.28760

**Published:** 2022-09-03

**Authors:** Heeya Shah, Omer Usman, Habib Ur Rehman, Sharan Jhaveri, Chaithanya Avanthika, Kamran Hussain, Hamza Islam, Sailesh I.S.K

**Affiliations:** 1 Medicine, G.M.E.R.S. (Gujarat Medical Education and Research Society) Medical College and Hospital, Gandhinagar, IND; 2 Medicine, Services Institute of Medical Sciences, Lahore, PAK; 3 Medicine, Ziauddin Medical College, Karachi, PAK; 4 Medicine, Smt NHL (Nathiba Hargovandas Lakhmichand) MMC (Municipal Medical College), Ahmedabad, IND; 5 Medicine and Surgery, Karnataka Institute of Medical Sciences, Hubli, IND; 6 Medicine, Nawaz Sharif Medical College, Gujrat, PAK; 7 Research, Faisalabad Medical University, Faisalabad, PAK; 8 Medicine, Madras Medical College, Chennai, IND

**Keywords:** parkinson's disease, neurodegenerative diseases, therapy, dyskinesia, deep brain stimulation

## Abstract

Parkinson’s disease (PD) is a common progressive neurodegenerative movement disorder. The cardinal feature of Parkinson's is neuronal degeneration causing a dopamine deficit in the brain which leads to a host of clinical features in the patient. However, consensus over specific clinical criteria for diagnosis remains to be established. Parkinson’s does not have a cure yet, but a variety of diagnostic and treatment protocols have been developed over the years with a primary focus on pharmacological therapy. Anti-parkinsonian drugs such as levodopa lose their efficacy over time and are needed in higher doses as the disease inevitably progresses. An alternative to pharmacological therapy is deep brain stimulation (DBS). Deep brain stimulation involves transcranial placement of unilateral or bilateral leads (wires) most commonly in the sub-thalamic nucleus or the globus pallidus interna of the brain by stereotactic surgery. Given the multiple hypotheses explaining the different effects of DBS with sometimes conflicting mechanisms, it is difficult to pinpoint the exact way in which DBS operates. Nevertheless, it has proven to be significantly effective. DBS, although being a cost-effective treatment measure for Parkinson's patients, is not without limitations. A careful selection of patients is required preoperatively that determines the response and tolerance to the therapy in patients. This review aims to summarize the current literature on DBS in Parkinson's with a focus on the hypothesized mechanisms, selection criteria, advantages and its limitations.

## Introduction and background

Parkinson’s disease (PD) is a common progressive neurodegenerative movement disorder affecting ~1%-3% of the global population over 60 years of age [1]. Symptoms include resting tremor, bradykinesia, rigidity, postural instability and a wide range of other motor and non-motor features are common [2]. Neurological deterioration is the cornerstone clinical feature; it largely manifests from the loss of the dopaminergic neurons of the substantia nigra pars compacta, which project axons to the striatum. Several ongoing studies have shown new insights concerning the pathophysiology of PD, suggesting that non-dopaminergic (ND) system is also affected and may correspond with multiple PD symptoms. The ND system includes several neurotransmitter and neuromodulatory systems within the basal ganglia and associated target areas, including glutaminergic, adrenergic, adenosine, serotonergic, histaminic, opioids and cholinergic pathways [3].

Increasing loss of dopaminergic neurons can be slightly compensated by dopaminergic substitutive therapy and therefore allow for symptom control, chiefly for motor symptoms. Simultaneously, ND medications have also been extensively investigated including adenosine A2A antagonists (istradefylline), glutamate antagonists (amantadine) and monoamine oxidase-B (MAO-B) inhibitors (selegiline) [3,4]. As the disease progresses, medical treatment becomes increasingly challenging. The development of motor fluctuations, the adverse effects of medical treatment or the appearance of therapy refractory motor symptoms act in concert to make the disease harder to manage. Also, these therapies are mainly symptomatic and do not change the disease course or treat the non-dopamine-dependent features of PD such as cognitive impairment, freezing of gait and other non-motor features of the disorder which often have a substantial influence on the quality of life [4].

For medication-refractory cases, deep brain stimulation (DBS) of the thalamic ventral intermediate nucleus (VIM) approved by FDA in 1997 was effective in treating essential tremors associated with PD [5,6]. Later, in 2002, FDA approved DBS which targeted globus pallidus interna (GPi) and sub-thalamic nucleus (STN) to treat levodopa-induced motor complications and advanced PD symptoms. And this evolved into one of the most effective surgical interventions [7]. DBS permits chronic and high-frequency stimulation of brain areas which have the equivalent effect of surgical ablation of these areas [8]. Additionally, it considerably reduces the postoperative usage of levodopa as a daily dose regime after STN DBS (up to 60%) and GPi DBS (up to 50%), thereby ameliorating the overall quality of life in patients with PD [9,10]. In certain parts of the world, because of its effectiveness, DBS is a promising treatment modality that is gaining popularity in the world for the treatment of advanced PD. Better outcomes, more flexibility, possible reversibility, less morbidity and lower mortality are among the few benefits of DBS [11]. This literature review focuses on the key insights and discusses the rationale of DBS in PD patients.

## Review

Mechanism of DBS 

**Figure 1:**
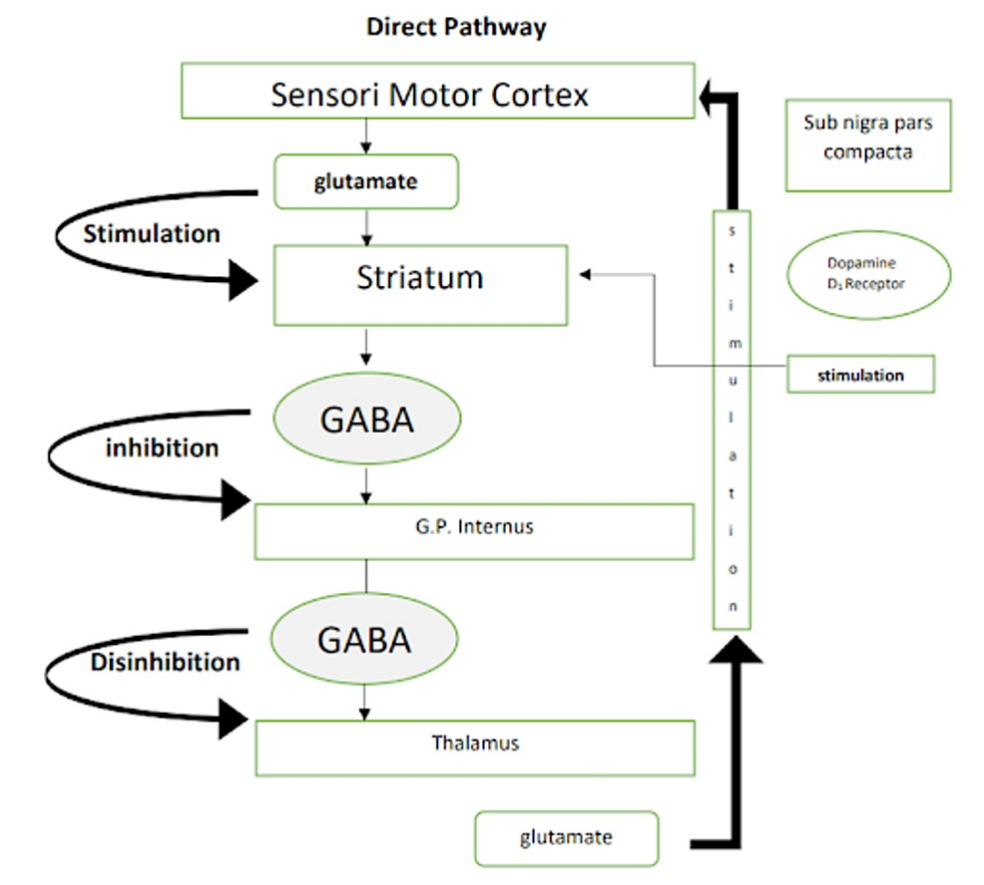
Pathways involved in Parkinson’s disease: direct pathway Image credits: Kamran Hussain. Sub nigra: substantia nigra, GABA: γ-aminobutyric acid, G.P.: globus pallidus.

**Figure 2:**
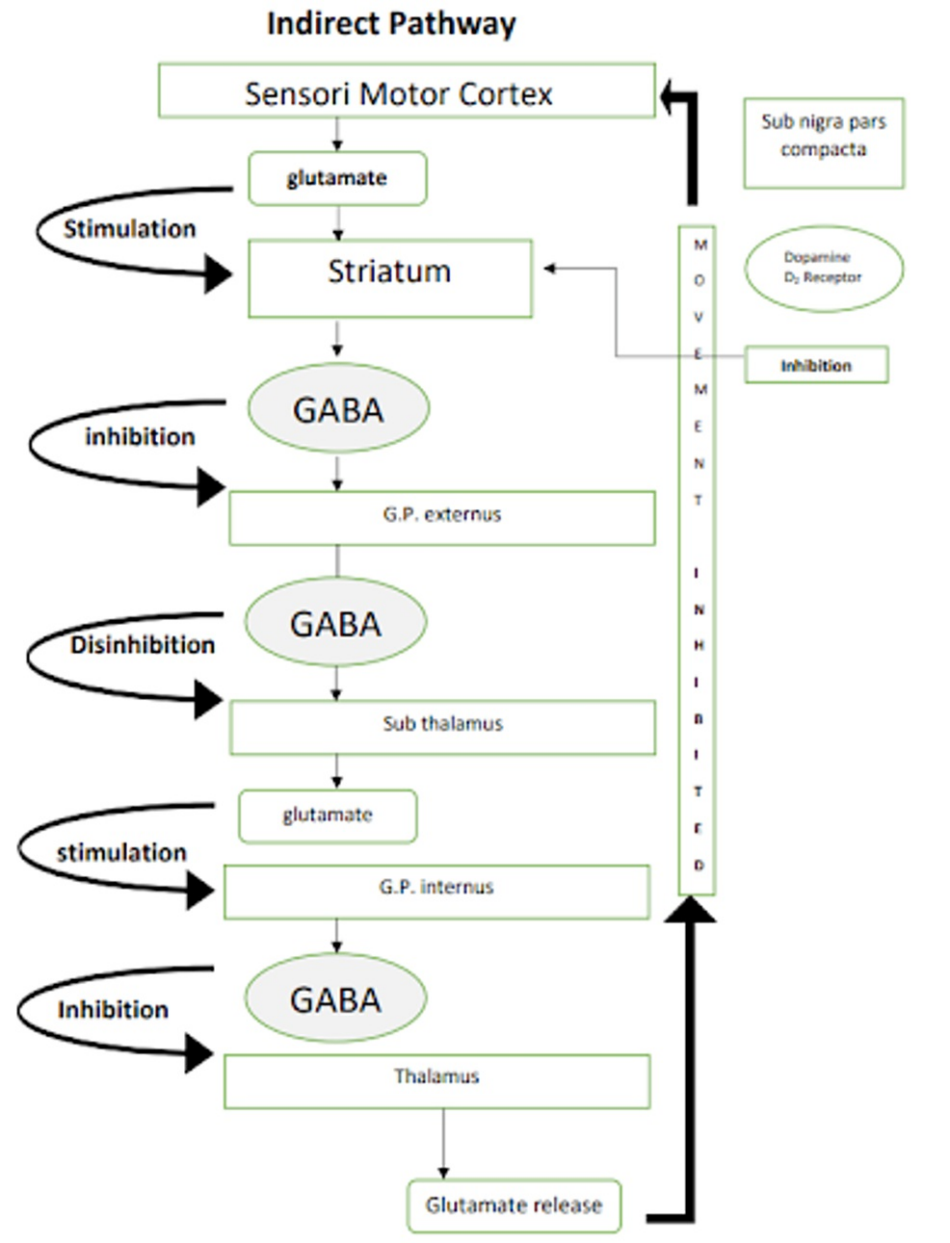
Pathways involved in Parkinson’s disease: indirect pathway Image credits: Kamran Hussain. Sub nigra: substantia nigra, GABA: γ-aminobutyric acid, G.P.: globus pallidus.

It is important to have a clear understanding of the normal physiology of the pathways of basal ganglia which are involved in Parkinson’s disease before we delve deeper into DBS. Figures 1, 2 are a simple representation of the same [12]. Pathways involved in Parkinson’s disease are shown in Figures 1, 2.

There are several hypotheses that try to define the mechanism of deep brain stimulation (DBS), but the exact mechanism is still uncertain. Deep brain stimulation involves transcranial placement of unilateral or bilateral leads (wires) in the STN or the GPi of the brain by stereotactic surgery. These leads are connected to a pulse generator in the chest that functions similarly to a pacemaker battery [13]. After surgical recovery, individuals who have undergone DBS join programmed visits to enhance adherence to parameters and medications. These electrodes generate electrical impulses, and data are recorded using amplifiers and electronic devices [14].

Low frequencies are those below 100 Hz, and high frequencies are those greater than 100 Hz. Lafreniere-Roula et al. performed an experiment on Parkinson diseased patients with the placement of electrodes through stereotactic surgery in GPi and substantia nigra pars reticularis (SNr) and studied the effect of DBS [14]. They found that high-frequency stimulation (HFS) (200 Hz) stimulated the γ-aminobutyric acid (GABAergic) afferent on these two locations, resulting in inhibition of GPi and SNr. Ultimately, disinhibition of the thalamus reduced the motor symptoms of parkinsonism [14]. Xu et al. experimented on monkeys and found similar results [15]. Boraud et al. performed a study on monkeys in three different states, i.e., normal state, after treatment with 1-methyl-4-phenyl-1,2,3,6-tetrahydropyridine (MPTP) and during HFS of GPi, and found similar results [16]. Benazzouz et al. studied the effect of HFS on the sub-thalamic nucleus (STN) of anesthetized rats and observed decreased neural firing causing disinhibition of thalamic motor nuclei and increased cortical motor firing [17].

On the other hand, some studies have shown the stimulatory effects of HFS. Hashimoto et al. experimented on two monkeys and studied the effect of MPTP and DBS [18]. They also did the histological analysis on monkeys’ brains. They concluded that DBS of STN increased the neuronal activity of GPi [18]. A study on rats by Windels et al. and a study on Parkinson diseased patients by Galati et al. showed that DBS of STN increased GABA in SNr and glutamate in globus pallidus (GP) resulting in increased neuronal activity in corresponding zones [19,20]. Hershey et al. observed with positron emission tomography (PET) scan that DBS of STN increased blood flow to STN, GP and thalamus and decreased blood flow to cortex [21]. Increased neurotransmitter level and blood flow in STN indicated increased neural activity in STN and stimulatory effects of HFS. Meanwhile, a study by Gale et al. showed that HFS increased dopamine release in caudate and putamen [22]. Therefore, it is difficult to pinpoint the exact way in which DBS operates, given the multiple hypotheses explaining the different effects of DBS with sometimes conflicting mechanisms.

Diagnosis and treatment of Parkinson’s 

Despite efforts to develop diagnostic tools or disease-specific biomarkers, Parkinson's disease (PD) is currently diagnosed by investigating typical clinical signs; there is no particular screening test. A thorough history and physical examination diagnose PD, and the history should include both motor and non-motor symptoms (Table 1) [23].

**Table 1:** Clinical features of PD PD: Parkinson’s disease.

Clinical features of Parkinson’s disease [1]	
Motor features	Non-motor features
Cardinal features	Hyposmia
Bradykinesia	Pain
Resting tremor	Sleep disturbances
Cogwheel rigidity	Sleep behavior disorder/insomnia
Postural instability	Daytime somnolence
Freezing of gait	Mood disturbance (depression)
Flexed posture	Cognitive impairment (loss of executive function)
	Gastrointestinal dysfunction (dysphagia, constipation)
	Genitourinary dysfunction (overactive bladder)
	Sweating dysfunction (hyperhidrosis or hypohidrosis)

A first-degree relative with PD increases the likelihood of being diagnosed with the condition [23]. Patients with PD must exhibit parkinsonism, which is defined as bradykinesia with resting tremor, cogwheel rigidity or both (Table 1) [24]. Individuals with a conclusive diagnosis of PD must also fulfill at least minimum two of the four supporting criteria: (1) resting tremor, (2) significant improvement with dopaminergic treatment (e.g., carbidopa-levodopa), (3) the existence of dyskinesias caused by levodopa and (4) the presence of olfactory loss or cardiac sympathetic denervation on iodine-123-meta-iodobenzylguanidine myocardial scintigraphy [24].

Dyskinesias are uncontrolled choreoathetoid movements caused by dopaminergic treatment. Around 50% of PD patients develop dyskinesias that arise nearly five years after starting pharmacotherapy. The medications are restricted if symptoms emerge [25]. In other circumstances, PD cannot be reported if drugs (antipsychotics, antidepressants) cause the patient's signs and symptoms or if enough results indicate an alternative diagnosis [24].

Dopamine transporter single-photon emission computed tomography (DaT SPECT) identifies presynaptic dopamine neuronal failure in PD and other neurodegenerative parkinsonisms by reduced absorption of a radioactive tracer that adheres to dopamine carriers in the basal ganglia. DaT SPECT shows high sensitivity and specificity in identifying nigrostriatal cell loss in PD patients [26]. DaT scans are typically useful only when the cause of parkinsonism on examination is uncertain. When a patient has evident parkinsonism, the scans are frequently positive and contribute nothing to the diagnostic picture [27]. They cannot identify PD from other Parkinson's disorders that entail dopamine transporter failure (e.g., multiple system atrophy) [27]. Magnetic resonance imaging (MRI) to diagnose PD is not recommended because MRI results might help differentiate PD from other parkinsonisms; improved methods may have diagnostic and predictive implications in the future [28]. A probable vascular contribution might be indicated by MRI findings of substantial cerebrovascular disease or basal ganglia [28]. Outside of the United States, iodine-123-meta-iodobenzylguanidine myocardial scintigraphy is commonly used to detect sympathetic nerve dysfunction, typically in PD patients [29]. These innovative technologies are now being studied as research tools, but they may one day be employed in regular clinical treatment. When paired with modern imaging methods, these revolutionary tools, cerebrospinal fluid analysis and skin and other tissue biopsies to check for synuclein disorders can help us detect PD sooner, even in the prodromal stage [30].

Conventional Medical Therapy

Initial treatments for PD symptoms include levodopa preparations, dopamine agonists and monoamine oxidase-B (MAO-B) inhibitors [13]. Anticholinergic drugs can benefit young persons with tremors, but they must be taken with extreme care due to the elevated risk of adverse effects, especially those impacting cognition. Levodopa-carbidopa enteral suspension is also used to treat motor fluctuations and dyskinesias [13].

Even though many clinicians avoid prescribing levodopa in the early stages of PD, the current study suggests that this is no longer applicable [31]. Individuals with PD often require more frequent levodopa doses, as well as greater doses over time (300-600 mg orally at least three times a day). It is not attributable to a decrease in drug tolerance or levodopa effectiveness. Individuals with PD lose their long-term response to dopaminergic treatment as the disease progresses, and their short-term response declines owing to disease-related pathophysiological modifications in the brain [32]. The brain cannot retain additional dopamine for future use [32].

We can combine several drugs with levodopa. In contrast to levodopa, which requires increasingly frequent dosage over time, MAO-B inhibitors and dopamine agonists are dosed nearly one to three times daily orally (1-3 mg) throughout the illness course. Catechol-O-methyltransferase inhibitors and MAO-B inhibitors suppress dopamine-digesting enzymes, prolonging the benefits of levodopa. In individuals with significant symptomatic interval and extended onset with consecutive dosage, inhaled levodopa can be utilized to produce a quicker drug response [33].

Use of DBS

DBS targets mainly the two main anatomic structures of the brain: sub-thalamic nuclear stimulation (STN-DBS) and globus pallidus internus stimulation (GPi-DBS). STN-DBS and GPi-DBS both improve motor function and activities of daily living in people with Parkinson's disease [34]. Neurostimulation of the sub-thalamic nucleus is proven to be more beneficial than medication alone [35]. Patients having DBS continue to require typical Parkinson's disease (PD) drugs but at a reduced dosage [36]. Much of the research has been on the post-operative response of non-motor symptoms, such as face recognition of PD patients. According to the findings, various responses accompany emotions such as joy and fear. For example, fear is accompanied by fight, flight, freeze or fawn responses, and joy is accompanied by smile or appreciation responses. DBS has been shown to exacerbate emotions associated with disgust [37]. Executive functions diminish as well over time. When it comes to non-motor symptom improvement, working memory improvement is a distinct outcome of DBS [38].

The DBS does not provide the same benefits to all persons suffering from Parkinson's disease in the same way. There is a plethora of elements that play a significant influence in the fact that not all patients receive the same benefits as others. Examples include unmotivated patients not following up on their appointments regularly, patients with critical medical comorbidities such as cardiovascular diseases, abnormal brain MRI scans, secondary parkinsonism and severe depression. All these factors are taken into consideration while developing exclusion criteria for DBS in PD [39]. A judicious selection of patients is essential to ascertain responsiveness and acceptability and to maximize the intervention's advantages (Table 2).

**Table 2:** Inclusion and exclusion criteria for DBS treatment DBS: deep brain stimulation.

	Patients to be excluded	Patients to be included
1	Severe psychosis	Absence of dementia or active psychiatry illness
2	Persistent depressive disorder	Levodopa motor complications despite optimal management
3	Levodopa non-responsive	Levodopa responsive
4	Atypical parkinsonism patient with dementia	Diagnosis of idiopathic Parkinson's disease (IPD)

Diagnosis of idiopathic Parkinson's disease (IPD)

Because of the vast range of PD symptoms and related behavioral difficulties, selecting patients for DBS has proven to be an incredibly hard assignment for treating physicians. The diversity of the clinical spectrum and the difficulty in capturing the clinical complexity of Parkinson's disease led to the development of standard assessment criteria to select patients for DBS known as the Core Assessment Program for Surgical Interventional Therapies in Parkinson's Disease (CAPSIT-PD). This includes the minimal prerequisite criteria for the evaluation of DBS in patients with Parkinson's disease. Most studies consider the factors influencing DBS acceptability and fatigability in sick populations. Thus, the re-evaluation of the Core Assessment Program for Surgical Interventional Therapies in Parkinson's Disease (CAPSIT-PD) should consider recent clinical trial advances and knowledge for surgical centers to effectively select the best target population and predict the best possible outcomes for patients [40]. To select the patient for DBS, a pre-procedural evaluation of mood and cognition is recommended (Table 3).

**Table 3:** To select the patients for DBS, pre-procedural evaluations of mood and cognition are recommended

Sr. No.	Procedure	Description	Example
1	Substantive procedural memory	Simple puzzles to assess the mental discipline	Short version of Tower of Hanoi
2	Precise memory	a) Verbal and visual memory testing	Visual amnesiac battery of Signoret
		b) Memory, attention and verbal learning ability testing	Rey Auditory and Verbal Learning Test (RAVLT)
3	Executive functioning skills	a) Working memory testing	Modified Brown Peterson Paradigm (MBPP)
		b) Auditory cognition function testing	Paced Auditory Serial Addition Test (PASAT)
		c) Linguistic rhythm testing	Verbal fluency: Letters F, A and S (FAS)

For the patient to have reasonable expectations from the treatment at the pre-procedural visit, certain motor symptoms and many non-motor symptoms, such as neuropsychiatric symptoms and cognition level, must be discussed with the neurologist before the procedure [41].

Cost-effectiveness and comparison of DBS to standard treatment protocols

Deep brain stimulation (DBS) is more cost-effective than other modes of treatment [42]. Although DBS does not provide a complete cure for PD, it does help in resolving symptoms and results in an overall better lifestyle among patients for up to 10 years [43]. The surgery for deep brain stimulation (DBS) is more cost-effective compared with the standard modes of drug therapies in PD [44]. In a study conducted in the United States by neurophysicians, DBS was compared with pharmacotherapy for PD in patients with an average age of 60, and the incremental cost-effectiveness ratio (ICER) was taken out based on dollars per quality-adjusted life-year (QALY). It was found that the treatment for DBS resulted in a total discounted cost of $130,510 compared to the discount for pharmacotherapy which was $91,026 per QALY over the period of 10 years [42]. Another study conducted in the United Kingdom compared the cost-effectiveness of DBS treatment to basic medical therapy interventions, and it was discovered that DBS treatment resulted in a mean cost per patient of £26,799 compared to basic medical therapy (£73,077/patient with basic medical therapy vs £46,278/patient with DBS). Additionally, a mean of 1.35 quality-adjusted life-years (QALYs) (6.69 QALYs with basic medical therapy versus 5.35 QALYs with DBS) yielded an increased cost-effectiveness ratio with DBS treatment with a 99% probability of DBS being cost-effective in parkinsonism [45,46].

This shows that DBS is quite cost-effective than standard pharmacotherapy in patients with motor complications providing them with long-term benefits [45]. A Swedish study using the Markov model also extracted the cost-effectiveness of DBS compared to medical treatment for PD. The cost-effectiveness ratio of DBS to medical treatment using duodopa and apomorphine was calculated. The results showed a net saving of Swedish Krona (SEK) 387,313 per QALY gained over 15 years [47]. In a randomized control trial, DBS treatment for PD patients was found to be more efficacious in improving motor symptoms like dyskinesias as compared to medical therapy [48]. DBS cost-effectiveness is associated with an improvement in the quality of life of PD patients around 18% when compared to those patients who were receiving medical treatment on the basis of incremental cost-effectiveness (C/E) ratio [49].

Risks and limitations of DBS

DBS is considered only when the symptoms are disabling despite optimal medical therapy. Optimal target localization can cause a significant reduction in the chances of side effects [50]. Hypertension is a significant risk factor for intracranial hemorrhage in PD patients undergoing DBS [51]. In a study conducted by Xiaowu et al., the incidence of hemorrhage in hypertensive patients was found to be more than twice that of their normotensive counterparts [52]. Microelectrode trajectories used in DBS also seem to increase the risk of intracranial hemorrhage [52].

In a study conducted by Schuepbach et al., the neurostimulation group and the patients in the medical therapy group were compared and the study concluded that depression was more frequent in the neurostimulation group [53]. The study also noted significant adverse effects related to surgery including cerebral abscess and non-specific edema. Surgery in the neurostimulation group also resulted in risks related to the device such as displacement leading to reoperation where displacement can be due to the dislocation of the stimulator, cable or lead [53].

DBS also increases the likelihood of risks such as suicide and hospital readmission with worsening of mobility or infection [54]. Progressive weight loss due to increased energy loss is seen in PD patients. Post-DBS of sub-thalamic nucleus (STN), weight gain has been reported [55]. This increase in weight may be associated with decreased energy loss due to the reduction in the tremor. The postoperative body weight gain may induce critical metabolic disorders. This demands the need for the management of body weight in PD patients undergoing DBS [56,57].

Tripoliti et al. studied the short-term and long-term speech response to STN-DBS patients to identify the clinical and surgical factors which are associated with speech change [58]. They came to the conclusion that both medical and surgical issues significantly contribute to speech decline in STN-DBS patients which can limit its beneficial effects. The reason for dysarthria and dysphagia can be attributed to the stimulation of STN and its vicinity to the corticobulbar pathways [57,58].

The connection of the sub-thalamic nucleus to the frontal cortex and basal ganglia structures forms a circuit that provides the basis for the mood changes including anger, post-DBS [59]. Evaluation of pre- to post-DBS revealed that STN and GPi DBS are associated with increased anger scores. These score changes were correlated to microelectrode passes suggesting it may be due to a lesional defect rather than a brain target-specific defect. Patients with a longer duration of disease were particularly susceptible to post-DBS anger compared to a shorter duration of the disease [60].

## Conclusions

DBS is used in the treatment of many diseases, including Parkinson’s. Despite being available for more than 25 years, its exact mechanism is yet to be established. Despite its many shortcomings, DBS has shown to be effective in patients where conventional medical therapy has failed. Although being a cost-effective treatment measure for Parkinson's patients, it is not without limitations. Establishing the exact mechanism of DBS as well as objectively quantifying the impact of DBS on Parkinson's patients is the need of the hour.
